# Augmenting Tumor‐Starvation Therapy by Cancer Cell Autophagy Inhibition

**DOI:** 10.1002/advs.201902847

**Published:** 2020-01-27

**Authors:** Bowen Yang, Li Ding, Yu Chen, Jianlin Shi

**Affiliations:** ^1^ State Key Laboratory of High Performance Ceramics and Superfine Microstructure Shanghai Institute of Ceramics Chinese Academy of Sciences Shanghai 200050 P. R. China; ^2^ Center of Materials Science and Optoelectronics Engineering University of Chinese Academy of Sciences Beijing 100049 P. R. China

**Keywords:** autophagy, black phosphorus, glycolysis, nanomedicine, tumor‐starvation therapy

## Abstract

It was recently recognized that cancer therapeutic efficacy may be greatly compromised by an intrinsic protective mechanism called autophagy, by which cancer cells survive in harsh conditions such as starvation. Here, a synergetic strategy is described for cancer treatment by suppressing such a protective mechanism for augmenting tumor‐starvation therapy. The synergetic therapy is achieved by restraining glucose metabolism using an antiglycolytic agent to predispose cancer cells to severe energy deprivation; concurrently the downstream autophagic flux and compensatory energy supplies are blocked by the autophagy inhibitor black phosphorus nanosheet. Cancer cells fail to extract their own nutrient to feed themselves, finally succumbing to therapeutic interventions and starving to death. Both in vitro and in vivo results evidence the cooperative effect between the autophagy inhibitor and antiglycolytic agent, which leads to remarkable synergetic antineoplastic outcome. It is expected that such a combinational approach by concurrently blocking exogenous and endogenous nutrition supplies will be beneficial to the design of effective tumor‐specific cancer therapies in the future.

## Graphical abstract

A synergetic cancer therapeutic concept is proposed by suppressing protective autophagy for augmenting tumor starvation therapy. This synergistic treatment is achieved by restraining glucose metabolism to predispose cancer cells to energy deprivation; concurrently the downstream autophagic survival is further blocked by the black phosphorus nanosheet, leading to a remarkable tumor‐suppression effect, as demonstrated both in vitro and in vivo.

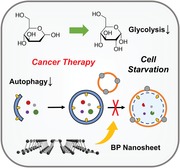

Autophagy is an intracellular catabolic process in which cells progressively capture cytoplasmic components and deliver them to lysosomal compartments for degradation.^1^ It is fundamental to the maintenance of intracellular homeostasis in physiological settings, as constitutive autophagic responses mediate the removal of potentially dangerous constituents and provide anabolic and catabolic substrates for supporting metabolism.^2^ Moreover, inducible autophagic responses also promote the normalization of the activities of cells encountering various extrinsic physicochemical perturbations, such as starvation,^3^ oxidative stress,^4^ hypoxia,^5^ hyperthermia,^6^ etc., underlying mechanisms that protect organisms against various pathological situations, including cancer, neurodegeneration, infection, and cardiovascular disease.^7^

However, neoplastic cells are also able to harness autophagy to survive under extreme adverse microenvironmental conditions, and to resist therapeutic challenges.^8^ A typical paradigm is tumor‐starvation therapy developed recently, in which pathways for the metabolism of cellular essential nutrition are blocked to predispose cancer cells to severe energy deprivation and starvation.^9^ To compensate for the nutritional and metabolic deficiencies, cancer cells elevate autophagy levels to “eat” and “digest” their own cytoplasmic components, providing additional avenues of metabolic support for re‐establishing homeostasis. For example, the generation of amino acids from autophagic protein degradation fuels biosynthesis and essential metabolic pathways, including tricarboxylic acid (TCA) cycle, and so ameliorates starvation.^10^ This intrinsic recycling function of autophagy promotes malignant cell survival under conditions of acute nutrient limitation, compromising the antineoplastic outcome of tumor‐starvation therapy. In line with this notion, cutting off the autophagic flux will become an effective therapeutic tool to retard the normalization of cancer cell metabolism and sensitize them to starvation therapy.^11^

Nanomedicines hold great promise for cancer management arising from the diverse unique physicochemical properties of nanomaterials.^12^ In general, nanomedicine internalization will provoke mild cytoprotective autophagic responses as these extraneous particles may influence intracellular homeostasis.^13^ Notably, several nanomaterials, such as nanodiamonds^14^ and titania‐coated gold nanoparticles,^15^ have been demonstrated to be capable of inhibiting autophagy via multiple pathways. A recent report indicates that black phosphorus (BP) nanosheets, which have been explored as robust biodegradable therapeutic platforms in various biomedical settings,^16^ could block autophagy flux and aggravate aberrant autophagosomes accumulation.^17^ The fast degradation of BP nanosheets in cancer cells as a consequence of intensified reactive oxygen species (ROS) production, promotes the acute elevation of intracellular phosphate anions (PO_4_^3−^). It has been well documented in inorganic chemistry that PO_4_^3−^ is alkaline in aqueous solution according to the Brønsted–Lowry acid–base theory (acidity coefficients of H_3_PO_4_: p*K*_a1_ = 2.15, p*K*_a2_ = 7.20, p*K*_a3_ = 11.90).^18^ As a result, the degradation of BP and subsequent PO_4_^3−^ accumulation in lysosomes will deplete the internal hydrogen ions by generating conjugated acid anions (HPO_4_^2−^), thus making the pH value deviated from that required for the most efficient action of hydrolases, which is believed to be responsible for the impairment of lysosomal functions in the process of autophagy. Therefore, BP nanosheets is therapeutically applicable to act as autophagy inhibitors and capable of potentiating tumor‐starvation therapy, by interrupting “self‐digest” of malignant cells during acute starvation.

While coping with the downstream disposal of autophagic substrates, a second consideration is the selection of adequate strategy that triggers upstream starvation. Cancer cells have adopted a metabolic switch to aerobic glycolysis, a phenomenon known as “Warburg effect.”^19^ The dependency on glycolysis proposes strategies by which such a metabolic phenotype is restrained to exacerbate energy depletion for antineoplastic effect. 2‐Deoxy‐d‐glucose (2DG), a glucose analog in which the C‐2‐hydroxyl group is replaced by hydrogen, has been extensively investigated as an antiglycolytic agent in clinical trials (Phase I/II).^20^ In this work, 2DG will be used in conjunction with BP nanosheets to confer primary pharmacological induction of starvation by retarding glycolysis. Consequent autophagic responses and compensatory energy supplies are further blocked in the context of BP nanosheet treatment. Cancer cells fail to extract their intrinsic nutrient to feed themselves, finally succumbing to therapeutic interventions and starving to death (**Scheme** 1). Both in vitro and in vivo data indicates that such a combinational therapeutic approach results in remarkable superadditive (“1 + 1 > 2”) antineoplastic effects, which may be instructive to the design of treatment regimens in the future.

**Scheme 1:**
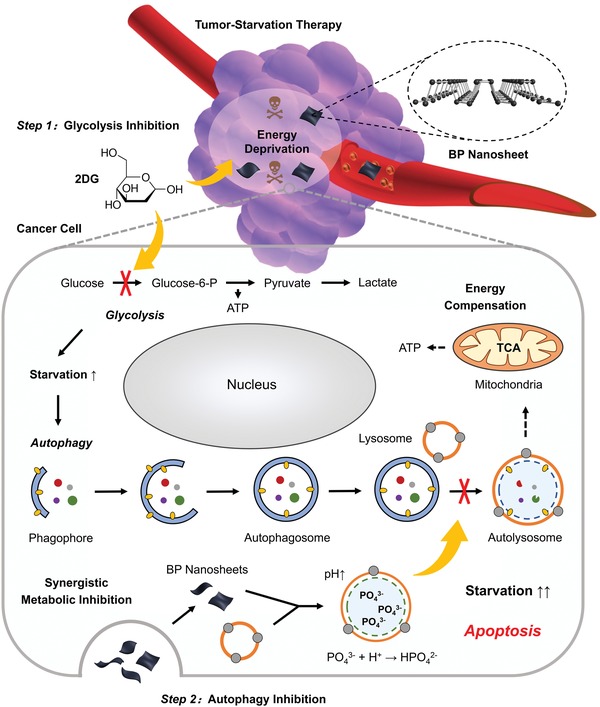
Schematic illustration for the mechanism of autophagy inhibition‐augmented tumor‐starvation therapy. Step 1: 2DG is applied to restrain glycolysis and initiate severe starvation of cancer cells. Step 2: BP nanosheet inhibits downstream protective autophagy, cuts off the compensatory nutrition supply and finally promotes apoptosis.

2D nanomaterials have been extensively explored as cancer therapeutic platforms recently.^21^ The ultrathin BP nanosheets were prepared according to a simple liquid exfoliation technique reported by previous literature.^22^ Transmission electron microscopy (TEM) image and scanning electron microscopy (SEM) image manifest that the as‐obtained BP nanosheets are free standing with lateral sizes of less than 200 nm (**Figure** 1a; Figure S1, Supporting Information), which is in line with the requirement for cellular uptake.^23^ Fourier transformation (FFT) image of a single BP nanosheet indicates its single crystalline structure with [010] preferential orientation, while the inverse fast FFT images at low and high magnifications further present its crystal lattice structure (Figure 1b,c). Theoretically, due to the layer‐structured nature of BP nanosheets (Figure 1d), the atomic arrangement projected along [010] direction is supposed to be amenable to the crystal lattice illustrated in Figure 1e, where the nearby two unbonded P atoms belong to two adjacent puckered P layers (S_2_ *_n_* and S_2_ *_n_*_+1_). However, in Figure 1c P atoms are arranged in agreement with the matrix in Figure 1f (S_2_ *_n_* alone), indicating that the investigated subregion in BP nanosheet is single layered. Atomic force microscopy (AFM) image and the cross‐sectional analysis show the distinct 2D topographic morphology of BP nanosheets with thicknesses of less than 1.6 nm (Figure S2, Supporting Information), from which it can be further concluded that the layer numbers of BP nanosheets are less than 4 according to the interlamellar spacing *d*_y_ = 5.4 Å (Figure 1d). These results provide robust evidences to demonstrate the ultrathin planar structure of as‐prepared BP nanosheets, which is closely associated with their high reactivity in oxidative aqueous environment,^24^ as it provides abundant anchoring sites to guest reactive molecules (e.g., ROS).^25^

**Figure 1:**
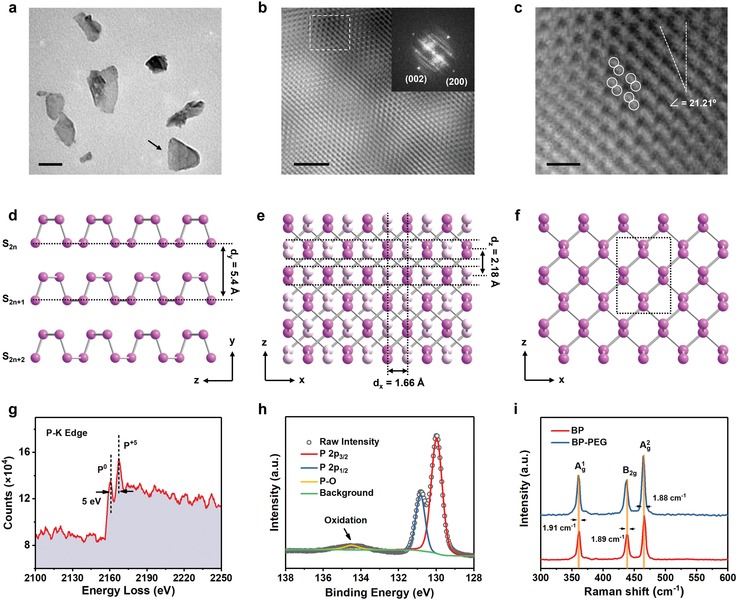
Characterizations of BP nanosheets. a) TEM image of BP nanosheets. Scale bar, 100 nm. b) Inverse fast FFT image of a single BP nanosheet marked in (a). Scale bar, 2 nm. Inset is the corresponding FFT image. c) Selected magnified inverse fast FFT image of dotted square region in (b). Scale bar, 0.4 nm. d–f) Schematics for the ideal atomic arrangement of multilayered (d,e) or monolayer (f) BP nanosheet along the [100] (d) and [010] (e,f) directions. The white atoms in (e) represent the second P‐layer behind the first one. The spatial conformation of a subunit in (f) corresponds to that of the marked atoms in (c). g) P K‐edge EELS of a single BP nanosheet marked in (a). h) P2p spectrum of XPS spectra for BP nanosheets. i) Raman spectra of BP nanosheets before and after PEGylation.

The chemical composition of as‐prepared BP nanosheets was further characterized by electron energy loss spectroscopy (EELS) and X‐ray photoelectron spectroscopy (XPS). As presented in Figure 1g, two significant signals of P K‐edge EELS are visible at the energy losses of 2160 and 2165 eV, respectively, which indicates the coexistence of P elements at the valences of 0 and +5. As is known, surface oxidation is an inevitable issue for BP nanosheets during the processes of liquid exfoliation and storage, and the generated PO*_x_* layer is structurally stable in anhydrous conditions that prevents the inner BP crystal from the further attack by ambient O_2_.^24^ The characteristic peaks at 129.9 and 130.9 eV in XPS spectra are related to the 2p3/2 and 2p1/2 orbitals of zero‐valent P in BP nanosheets, while the weak peak at 134.5 eV corresponds to PO*_x_* species on the surface of the nanosheets (Figure 1h).

It is noted that such a minor oxidation layer is unstable in aqueous solution and phosphate anions will be generated after reaction with H_2_O, leading to the distinct decrease of zeta potential of the whole system (Figure S3a, Supporting Information). Due to the electron‐screening effect, BP nanosheets would aggregate in saline solution environments such as biological milieu.[qv: 16b] To improve their biostability for favoring subsequent therapeutic applications, BP nanosheets were modified with PEG‐NH_2_ based on the electrostatic‐adsorption principle. The zeta potential of BP nanosheets increases significantly from −25.8 to −4.3 mV after PEGylation (Figure S3a, Supporting Information), while the characteristic stretching vibrations at 2887 and 1089 cm^−1^ in Fourier transform infrared (FTIR) spectra are attributed to –CH_2_– and C–O–C of PEG on BP nanosheets (Figure S3b, Supporting Information). A slight band shift in Raman spectra toward a low wavenumber can also be observed, which results from a slight increase in the thickness of BP nanosheets after PEGylation (Figure 1i). These engineered BP nanosheets exhibit high dispersity in various solvents including H_2_O, phosphate buffer solution (PBS) and Dulbecco's modified Eagle medium (DMEM), and no noticeable precipitation can be observed in 2 h of dispersion (Figure S3c, Supporting Information). These results manifest the successful engineering of PEG‐NH_2_ on BP nanosheets, which guarantees their in vivo physiological stability and therapeutic performance. In the following text, the word BP nanosheets is used throughout this article to refer to PEGylated BP nanosheets for a simplified elucidation, unless otherwise specified.

The prepared BP nanosheets should work in with adequate strategies that trigger upstream cancer‐cell starvation to elicit optimal synergy. The long‐dismissed evidence that cancer cells have specific metabolic alterations can be harnessed for the development of efficient therapeutic approaches. To meet increased bioenergetic demands for growth and proliferation, malignant cells are prone to catabolizing glucose followed by lactate fermentation in cytosol even under sufficient oxygen tension, rather than by mitochondrial oxidative phosphorylation (OXPHOS).^19^ In fact, glycolysis is a less efficient metabolic phenotype for adenosine 5′‐triphosphate (ATP) generation (2 ATP per metabolized glucose) compared with OXPHOS (36 ATP per metabolized glucose). Nevertheless, the rate of ATP production from the former pathway is much more adjustable and ≈100‐fold faster than that from the latter one.[qv: 20b] As a consequence, to achieve rapid energy supply during malignant proliferation, cancer cells consume at least ten times more glucose than normal cells^26^ and increase the rate of glycolysis by over 30 folds.[qv: 20b]

This hallmark has fostered the development of approaches to target glycolysis for antineoplastic therapy. 2DG, a synthetic glucose analog in which the C‐2‐hydroxyl group is replaced by hydrogen, has been widely used as a model antiglycolytic agent to study reduced metabolic activity of cancer cells.[qv: 20b] Due to the structural similarity to glucose, 2DG can also be actively taken up by the glucose transporters (GLUTs) and thus competitively inhibits glucose uptake.[qv: 20b] In cytoplasmic environment, 2DG is phosphorylated by hexokinase (HK) to form 2DG‐6‐phosphate, which cannot be further metabolized but accumulates and noncompetitively inhibits glucose phosphorylation.^27^ The two events lead to decreased ATP production from glycolysis, predisposing cancer cells to severe energy deprivation.^28^ Therefore, 2DG is competent to trigger upstream tumor starvation, and we expect that 2DG may operate synergistically with BP nanosheets for optimal therapeutic effect.

To validate our hypothesis, the effects of 2DG, BP nanosheets and synergistic therapy (2DG + BP) on glycolysis were first investigated at cellular level. Human malignant melanoma cell line A375 and human cervical cancer cell line HeLa, were selected to be incubated with different concentrations of 2DG and BP nanosheets either alone or in combination. As a characteristic metabolite of glycolysis, the extracellular lactate was observed with significantly decreased production both from A375 and HeLa cells after 2DG treatment (**Figure** 2a). Either elevating 2DG concentrations or prolonging 2DG incubation durations could further aggravate the blockage of lactate production (maximum 77.6% inhibition). Comparatively, BP nanosheets alone failed to elicit distinct inhibitory effect, although at a high BP concentration (64 ppm) the production of lactate from HeLa cells decreased slightly (16.79%), which may result from the intrinsic cytotoxicity of BP.^17^ These results demonstrate that 2DG is efficient in restraining glycolysis, while BP nanosheets have minor (or even negligible at low concentrations) antiglycolytic effect. The actual effect of synergistic therapy on lactate production is close to the theoretical linear addition of the effects of single 2DG and BP, confirming that BP nanosheets work independently from glycolysis and have no collateral effect on 2DG‐involved glucose metabolism inhibition.

**Figure 2:**
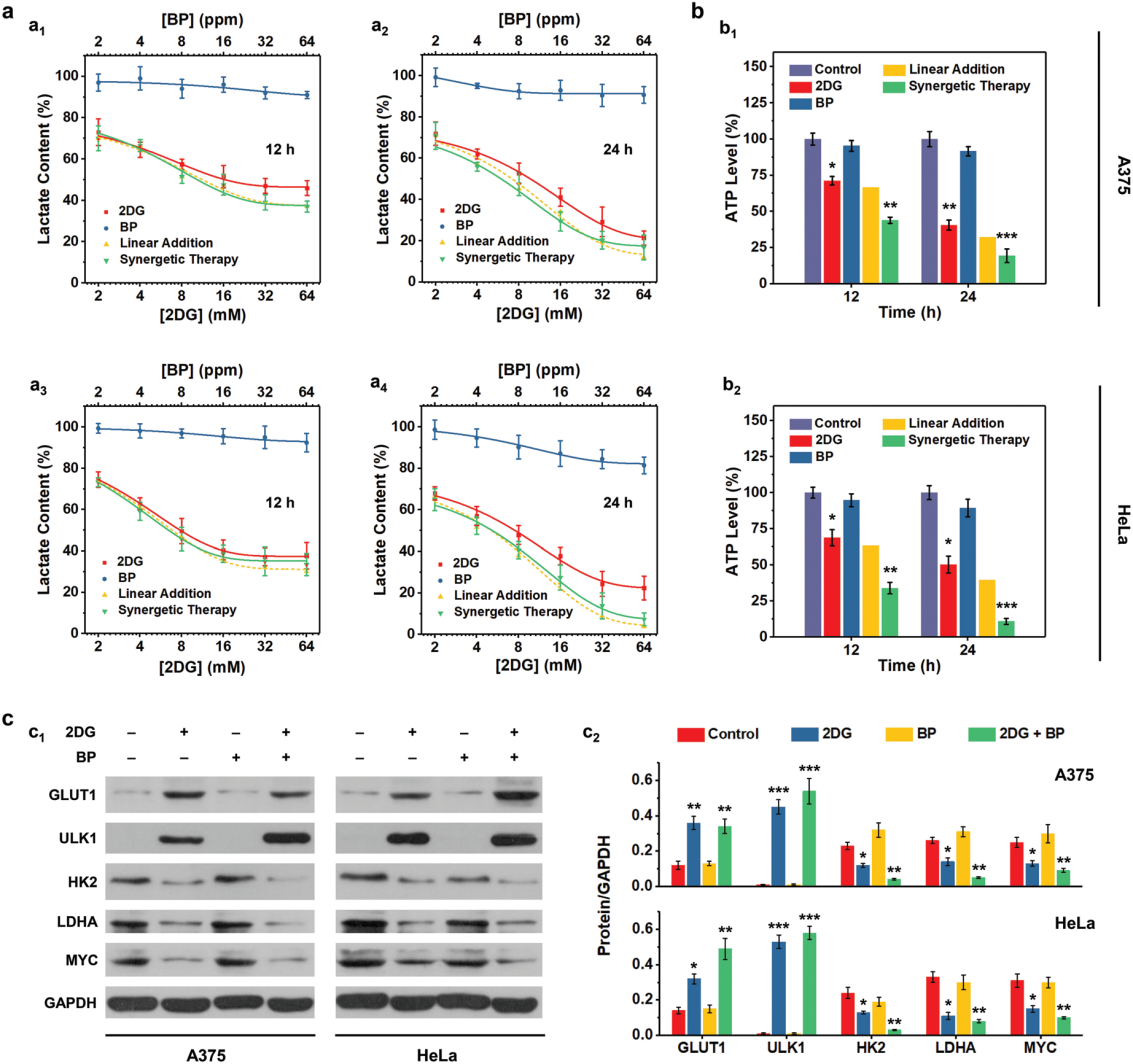
In vitro evaluation for glucose metabolism inhibition. a) Extracellular lactate contents of A375 (a_1_,a_2_) and HeLa cells (a_3_,a_4_) after various treatments for 12 (a_1_,a_3_) and 24 h (a_2_,a_4_) as indicated in the figure. Linear additions for the effects of single 2DG or BP treatment on lactate production are plotted for comparison with that of synergistic therapy group. Data are expressed as means ± SD (*N* = 6). b) Intracellular ATP levels of A375 (b_1_) and HeLa (b_2_) cells after various treatments as indicated in the figure. Linear additions for the effect of sole 2DG or BP treatment on ATP production are calculated for comparison with that of synergistic therapy group. Data are expressed as means ± SD (*N* = 3). c) Immunoblot analyses (c_1_) of GLUT1, ULK1, HK2, LDHA, and MYC expression levels in A375 and HeLa cells after different treatments for 24 h. Glyceraldehyde‐3‐phosphate dehydrogenase (GAPDH) expression levels serve as the loading controls. The corresponding quantitative data (c_2_) was also presented for comparison. Experiments were repeated three times with similar results. Statistical significances were calculated via Student's *t‐*test. *P < 0.05, **P < 0.01, and ***P < 0.001.

As discussed in the previous section, the generation of ATP in malignant cells is mainly from, but not restricted to glycolysis, as other metabolic pathways, such as TCA cycle also provide an alternate energy source.^19^ Moreover, inducible autophagic responses during starvation can also promote protein degradation and amino acid generation that feeds TCA cycle.^8^ To clarify the characteristics of energy metabolism, the intracellular ATP levels of A375 and HeLa cells were also quantified after treatment with single agent or their combination (Figure 2b). Distinct decreases of ATP production were observed in both two cell lines after 2DG treatment, especially in 24 h of incubation (59.4% for A375 and 49.7% for HeLa), while no significant change was observed in BP nanosheets groups, demonstrating that glycolysis inhibition alone by 2DG is able to exacerbate energy deprivation, and BP nanosheets cannot directly target energy metabolism as glycolytic activity has not been reduced. Importantly, the effect of synergistic therapy on suppressing ATP production is much more significant than the theoretical addition of those of monotherapies, suggesting that the synergistic therapy has yield superadditive effect on energy deprivation in cancer cells, which may be associated with the blockage of downstream autophagic flux by BP nanosheets that cut off the remedial energy supply through TCA cycle following glycolysis inhibition.

To further investigate the molecular mechanism of glycolysis inhibition, as well as the association between the pathways of glycolysis and autophagy, immunoblot analysis was conducted to assess the expressions of glycolysis‐related proteins in A375 and HeLa cells (Figure 2c). The levels of HK2, lactate dehydrogenase A (LDHA) and MYC were reduced in 2DG and synergistic therapy groups, evidencing the reduction of glycolytic activity.^29^ Comparatively, GLUT1 and UNC‑51‐like autophagy‐activating kinase 1 (ULK1) were overexpressed in the two groups, which may result from autophagy activation that upregulates GLUT1 expression and phosphorylates key glycolytic enzymes to compensate for the glycolysis inhibition by 2DG.^30^

The association between the pathways of glycolysis and autophagy encourages us to further investigate the downstream autophagic responses after different treatments, as well as their roles in re‐modulating energy metabolism. Cells were transfected with green fluorescent protein (GFP)‐tagged microtubule‐associated protein 1 light chain 3B (LC3B) pcDNA before treating with 2DG, BP nanosheets or their combination. Distinct GFP‐LC3B puncta with green fluorescence can be visualized in 2DG, BP, and synergistic therapy groups (**Figure** 3a), characteristic of the increase in the quantity of autophagosomes.^31^ However, these results are not sufficient to evidence whether autophagy is activated or inhibited in one specific scenario, as autophagosomes accumulate not only in the process of anabatic autophagic responses (increased on‐rate), but also when the formation and/or activity of autolysosomes is inhibited (decreased off‐rate).^32^ These cells expressing GFP‐LC3B were stained with LysoTracker (a dye specific for lysosomes) to further investigate the features of intact autophagic flux. Negligible colocalization of GFP‐LC3B puncta with LysoTracker‐stained subregions was observed in control group, as these cells are in basal levels of autophagy and few autophagosomes are fused with lysosomal compartments. Comparatively, GFP‐LC3B puncta were well colocalized with LysoTracker (i.e., yellow fluorescence) in 2DG, BP, and synergistic therapy groups, demonstrating the increasement of autolysosomes, which may result from either the initiation of productive autophagic responses or the blockage of lysosomal degradation.^32^

**Figure 3:**
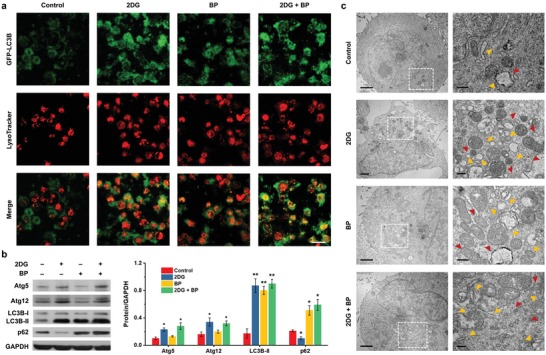
In vitro evaluation for autophagic responses. a) Confocal fluorescence images of GFP‐LC3B‐expressing A375 cells after various treatments. GFP‐LC3B puncta (green) reflect the formation or accumulation of autophagosomes, while the stained LysoTracker (red) was used to monitor the lysosomes. The colocalization of GFP‐LC3B puncta and LysoTracker (yellow) indicates the fusion between autophagosomes and lysosomes, and the consequent autolysosome formation. Scale bars, 50 µm. b) Immunoblot analyses and corresponding quantitative data of Atg5, Atg12, LC3B, and p62 expressions in A375 cells after different treatments for 24 h. GAPDH expression levels serve as the loading controls. Experiments were repeated three times with similar results. Statistical significances were calculated via Student's *t* test. *P < 0.05 and **P < 0.01. c) Bio‐TEM images of A375 cells after various treatments at low (scale bars, 2 µm) and high (scale bars, 0.5 µm) magnifications. The yellow and red triangle marks represent initial and degradative autophagic vacuoles, respectively.

Despite considerable efforts towards standardization, the fluorescence microscopy based on static autophagosomal and lysosomal biomarkers fails to provide concrete evidences in support of the differentiation between positive or negative modulations of autophagy, or even their combination. More comprehensive investigations at molecular level are required. As a complementary but indispensable method, immunoblot analysis was conducted to assess the expressions of autophagy‐related proteins (Figure 3b; Figures S4 and S5, Supporting Information). Beclin 1, Atg 5, and Atg 12 were upregulated both in 2DG and synergistic therapy groups, indicating accelerated autophagosomal processing.^33^ As a typical autophagosomal biomarker, the upregulation of LC3B‐II in 2DG, BP, and synergistic therapy groups suggests the increasement of autophagosome quantity, which is in agreement with the result of fluorescence microscopy. More importantly, p62, a specific substrate recruited to autophagosomal membrane and degraded in autolysosomes,^34^ was evidenced to accumulate in BP and synergistic therapy groups, demonstrating the blockage of lysosomal degradation. Comparatively, this autophagic substrate was downregulated in 2DG group, which reveals the boosting of autophagic flux after starvation treatment. These results provide solid evidences to discriminate the situations between increased on‐rate and decreased off‐rate, i.e., 2DG provokes and accelerates autophagic process, BP nanosheets block lysosomal function, synergistic therapy group promotes the upstream autophagosomal processing but inhibits downstream lysosomal degradation.

Such an elucidation was further confirmed by flow cytometry and bio‐TEM. DAPGreen, a fluorescent probe that can emit strong fluorescence in the membrane of autophagosome and early autolysosome,^35^ was applied in flow cytometry to provide quantitative information (Figure S6, Supporting Information). Elevated concentrations of 2DG and BP enable the progressive enhancement of DAPGreen fluorescence intensity, demonstrating that the accumulation of autophagic machinery positively correlates with the dosage of 2DG/BP. Furthermore, the fluorescence intensity in synergistic therapy group is higher than that in 2DG group, indicating that BP nanosheets do not antagonize or counteract the effect of 2DG on autophagy, but work independently and cooperatively that exacerbate the accumulation of autophagic machinery and substrate. To observe the autophagic flux in developmental scenarios, bio‐TEM was also used (Figure 3c; Figure S7, Supporting Information). Compared with cells in control group, which are in the courses of constitutive autophagic responses, more initial and degradative autophagic vacuoles (AVi and AVd, respectively) were observed in 2DG‐treated cells, manifesting boosted autophagic flux. The accumulation of AVi and AVd could also be visualized in BP group, as the consequence of blockage in the disposal of autophagosomes and autolysosomes. Importantly, the numbers of AVi and AVd increase distinctly in synergistic therapy group, which is due to the cooperative effect between upstream autophagy activation and downstream autophagy inhibition.

The mechanism of autophagy inhibition by BP nanosheets was further explored via the measurement of intracelluar pH of the two cancer cell lines, which indicates slight elevations of the pH values due to the generation of alkaline PO_4_^3−^ by BP nanosheets after interacting with intracelluar excessive ROS (Figure S8, Supporting Information).^36^ As a consequence, the intracelluar H^+^ was depleted and the function of lysosome was restrained, finally the autophagic flux was blocked. Such an autophagy‐inhibition mechanism of BP nanosheet is analogous to that of the typical autophagy inhibitor chloroquine, which is also a weak base (p*K*_a1_ = 10.2, p*K*_a2_ = 8.1)^37^ and capable of depleting intracelluar H^+^ for blocking lysosomal hydrolases function.^38^ From the perspective of the whole autophagy process, BP nanosheets retard autophagic responses at the stage of early autolysosomes by inhibiting the degradation of autophagic substrates, which cuts off the flux of nutrients into TCA, thus blocking the endogenous compensatory energy supply pathway during starvation. As a consequence, BP nanosheets may play a significant role in potentiating tumor starvation therapy by inhibiting autophagy and energy metabolism.

To evaluate the antineoplastic effect of synergistic therapy, A375 and HeLa cells were treated with different concentrations of 2DG and BP nanosheets either alone or in combination. The cell viabilities of different groups were systematically investigated by a standard Cell Counting Kit‐8 (CCK‐8) assay (**Figure** 4a,b). BP nanosheets had minor influence on the viabilities of A375 and HeLa cells, while 2DG alone could kill a certain percentage of cells, which is attributed to its distinct antiglycolytic potency. Of note, the reduction in cell viabilities is less significant than that in lactate production at the same 2DG concentration as presented in Figure 2a (61.6% vs 77.6% when [2DG] = 64 × 10^−3^ m), revealing that a proportion of cancer cells has survived by activating alternative metabolic pathways beyond glycolysis. Importantly, the effect of synergistic therapy on reducing cancer cell viabilities is much stronger than the theoretical addition of that of monotherapies, suggesting that BP nanosheets serve not as a standalone intervention but as an auxiliary means to sensitize malignant cells to starvation. Although 2DG alone is not sufficient for therapeutic purpose, as starvation provokes autophagic responses that promotes catabolism and counteracts nutritional deficiency,^39^ the employment of BP nanosheets is evidenced to be efficient in maintaining and aggravating starvation by inhibiting autophagy, thus significantly elevating the antineoplastic effect.

**Figure 4:**
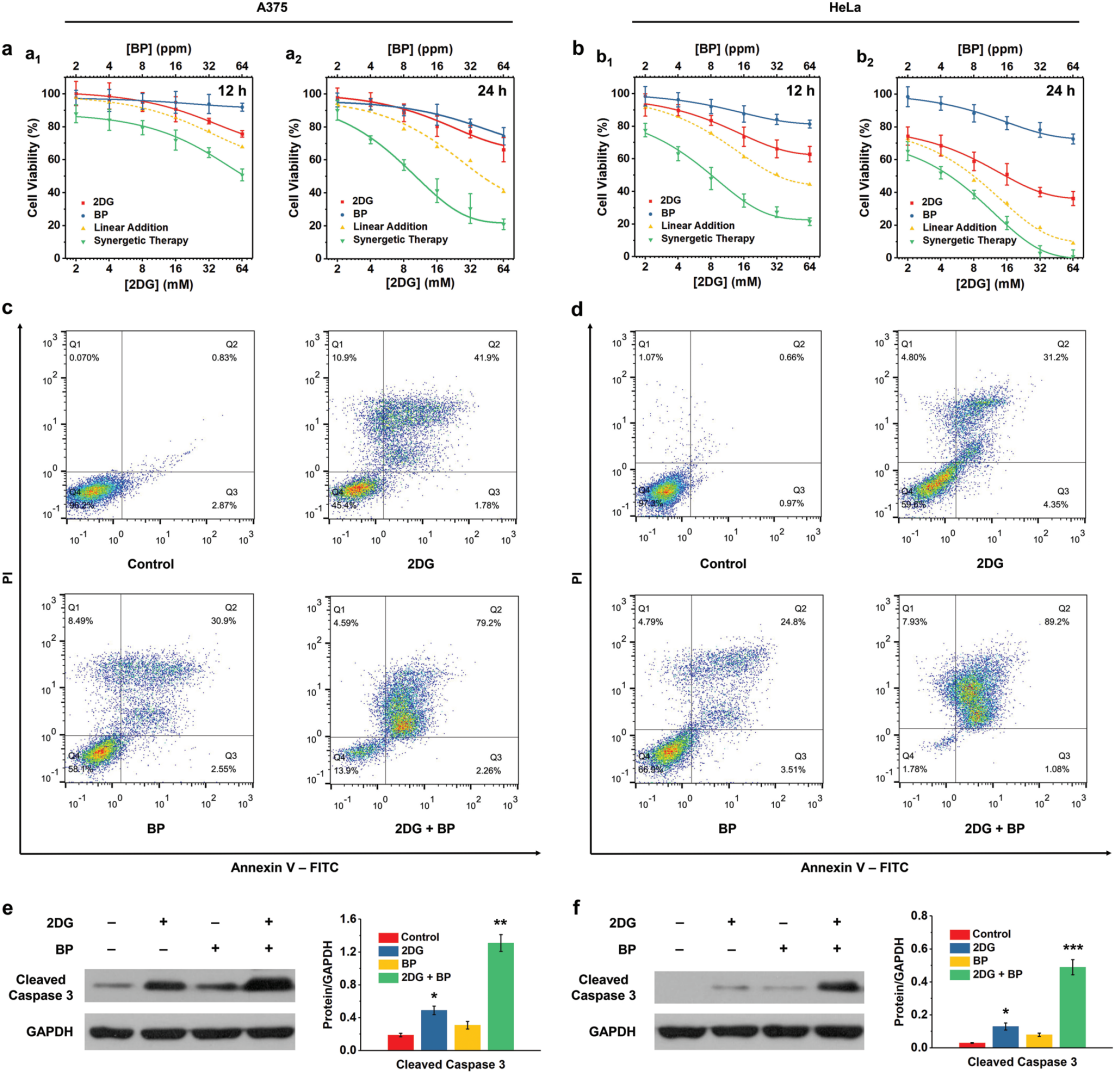
In vitro evaluation for apoptosis induction. a,b) Viability assays of A375 (a) and HeLa (b) cells after various treatments for 12 h (a_1_,b_1_) and 24 h (a_2_,b_2_). Linear additions for the effects of single 2DG or BP treatment on cell viability are plotted for comparison with that of synergistic therapy group. Data are expressed as means ± SD (*N* = 6). c,d) Flow cytometric analysis for the apoptosis of Annexin V‐FITC/PI‐strained A375 (c) and HeLa (d) cells after different treatments for 24 h. e,f) Immunoblot analyses and corresponding quantitative data of cleaved caspase 3 expression levels in A375 (e) and HeLa (f) cells after different treatments for 24 h. GAPDH expression levels serve as the loading controls. Experiments were repeated three times with similar results. Statistical significances were calculated via Student's *t‐*test. *P < 0.05, **P < 0.01, and ***P < 0.001.

The concrete cell death pathways were further investigated by flow cytometry and immunoblotting. A375 or HeLa cells in different groups were stained with fluorescein isothiocyanate (FITC)‐labeled Annexin V and propidium iodide (PI) for flow cytometric analysis (Figure 4c,d). Compared with the control group, in which nearly all cells were alive (Q1 quadrants, 96.2% for A375 and 97.2% for HeLa), a certain number of cells incubated with 2DG exhibited late apoptosis (Q2 quadrants, 41.9% for A375 and 31.2% for HeLa), revealing that glycolysis inhibition is able to guide cells to apoptotic pathways. BP nanosheets could also lead a number of cells to late apoptosis (Q2 quadrants, 30.9% for A375 and 24.8% for HeLa), which is attributed to its intrinsic cytotoxicity. Cells in synergistic therapy groups presented significantly elevated late apoptosis ratio (Q2 quadrants, 79.2% for A375 and 89.2% for HeLa) compared with other two experimental groups, further confirming that such a combined therapeutic strategy is efficient enough to elicit potent cell starvation and consequent apoptotic cell death. Moreover, immunoblot analyses indicated that the expressions of cleaved caspase 3 in synergistic therapy groups were significantly upregulated compared with those in 2DG and BP nanosheets groups (Figure 4e,f), further evidencing the apoptotic pathway of cancer cells after synergistic treatment.

Substantial cellular results on the synergy between starvation and autophagy inhibition lead us to further explore the therapeutic potential of the combinational therapy in vivo. Two xenograft tumor models were established by subcutaneously injecting A375 and HeLa cells into the axilla of four‐week‐old female Balb/c nude mice. When the tumor size reached about 100 mm^3^, A375‐ or HeLa‐tumor‐bearing nude mice were randomly divided into four groups: PBS (control), 2DG, BP, and 2DG + BP (synergistic therapy). During an observation period of 14 d, fast increase of the tumor volumes in control groups was visualized, while the growth of tumors in 2DG and BP groups was slightly restrained (**Figure** 5a,d; Figure S9, Supporting Information). It is noted that the antineoplastic effect of synergistic therapy is much more significant than the theoretical addition of those of monotherapies (76.97% vs 52.32% for A375, 79.16% vs 55.47% for HeLa), demonstrating that the blockage of autophagy by BP nanosheets is an efficient route to potentiate tumor starvation therapy, as a considerable extra therapeutic potential has been exerted through the combined approach. No significant weight fluctuations were observed in all groups (Figure 5b,e), confirming negligible adverse effects of these treatments. The life span of mice in synergistic therapy group was prolonged compared with other three groups (Figure 5c,f), thanks to the desirable therapeutic outcome of synergistic therapy.

**Figure 5:**
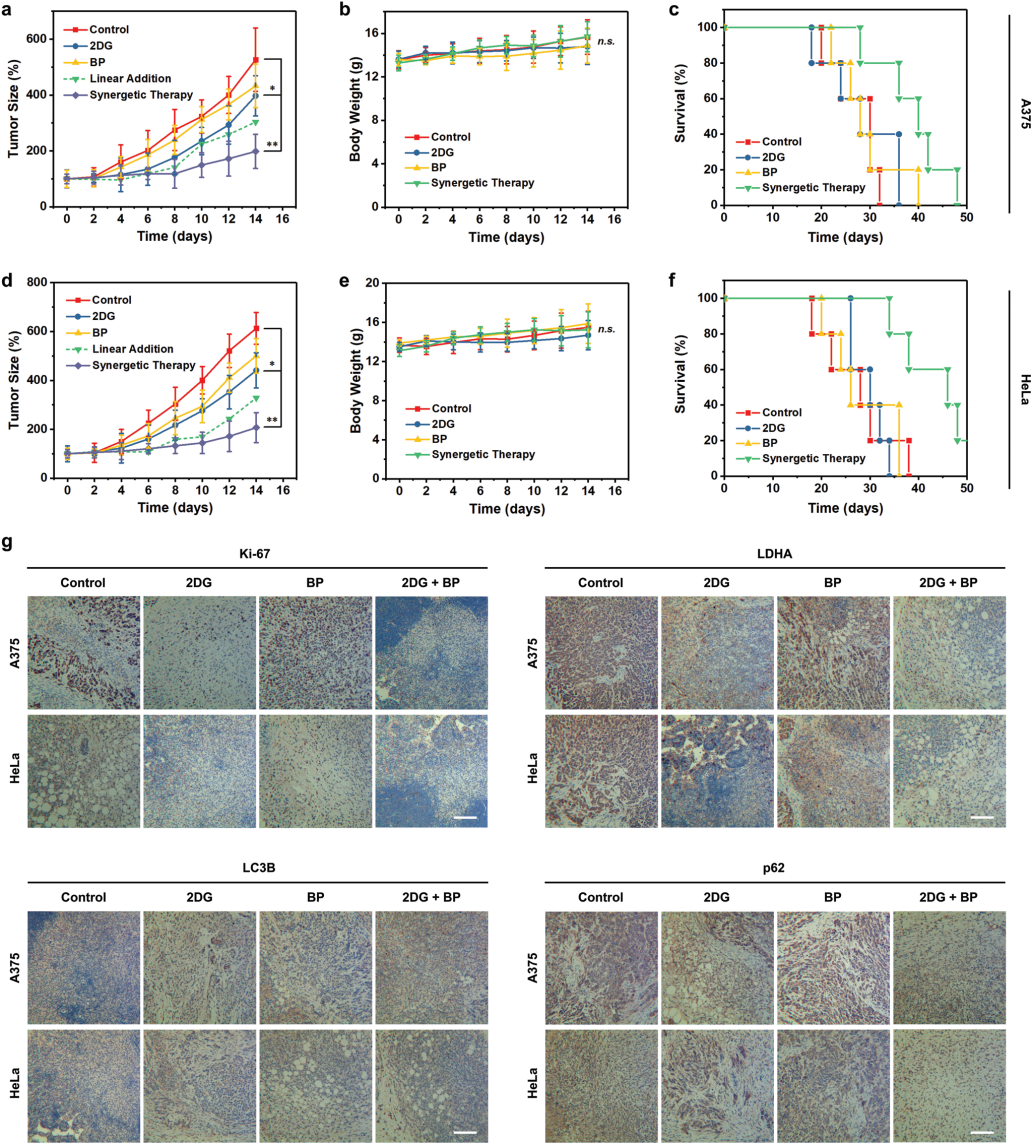
In vivo evaluation for antitumor therapy. a) Growth curves of tumor xenografts of A375 cells in mice treated with: (1) PBS (control); (2) 2DG; (3) BP nanosheets; (4) the combination of 2DG and BP (synergistic therapy). Error bars are based on SD (*N* = 5). b) Time‐dependent body‐weight curves of A375‐tumor‐bearing mice after different treatments. Error bars are based on SD (*N* = 5). c) Kaplan–Meier survival curves of A375‐tumor‐bearing mice after different treatments (*N* = 5). d) Growth curves of tumor xenografts of HeLa cells in mice after different treatments as indicated in (a). Error bars are based on SD (*N* = 5). e) Time‐dependent body‐weight curves of HeLa‐tumor‐bearing mice after different treatments. Error bars are based on SD (*N* = 5). f) Kaplan–Meier survival curves of HeLa‐tumor‐bearing mice after different treatments (*N* = 5). g) Immunohistochemical analyses of Ki‐67, LDHA, LC3B, and p62 of xenografted A375 and HeLa tumor sections after different treatments at day 14. Scale bars, 100 µm. Statistical significances were calculated via Student's *t‐*test. n.s., not significant. *P < 0.05, **P < 0.01.

To further confirm the mechanism responsible for therapeutic effects, immunohistochemical assays of Ki‐67, LDHA, LC3B, and p62 of xenografted tumor sections were also conducted (Figure 5g). The Ki‐67 level was significantly downregulated in synergistic therapy groups compared to other three groups, indicative of restrained cancer cell proliferation after the synergistic therapy. The expression of LDHA in 2DG and synergistic therapy groups was much lower than that in control and BP groups, demonstrating that glycolysis had been restrained after 2DG treatment. LC3B processing was upregulated in 2DG, BP, and synergistic therapy groups, revealing the increasement of autophagosomes in tumor cells after these treatments. Moreover, tumors treated with 2DG alone displayed reduced abundance of p62, consistent with boosted autophagic flux. Comparatively, the expression of p62 was upregulated in BP and synergistic therapy groups, indicating blocked lysosomal degradation. These immunohistochemical results are in agreement with the cellular level data that 2DG retards glucose metabolism and provokes productive autophagic responses, BP nanosheets block the degradation of autophagic machinery and substrates, and synergistic therapy inhibits glycolysis and autophagy, leading to acute starvation as well as consequent cell death. Moreover, immunofluorescent assays of terminal deoxynucleotidyl transferase dUTP nick end labeling (TUNEL), GLUT1, and LC3B of tumor sections also validated the conclusion (Figures S10 and S11, Supporting Information). In addition to histological evaluations of tumor sections, hematoxylin and eosin (H&E) staining of major organs (heart, liver, spleen, lung, and kidney) in different groups displayed no acute, chronic adverse effects (Figures S12 and S13, Supporting Information), further evidencing that these treatments are biocompatible.

Combinational tumor therapeutic strategies by harnessing autophagy to sensitize cancer therapies are under extensive investigations very recently. In this work, efforts are dedicated, for the first time, to using BP nanosheet as an autophagy inhibitor to augment tumor‐starvation therapy. Restraining glycolysis predisposes cancer cells to severe energy deprivation, but this operation also activates autophagic responses to “eat” their own components for survival. BP nanosheet blocks the autophagic flux and subsequent metabolic pathway, finally starving cancer cells to death. Both in vitro and in vivo results evidence the cooperative effect between BP and 2DG, which leads to remarkable superadditive therapeutic outcome as well as negligible side effect. Our follow‐up study is to implement orthogonal tests to optimize the proportion between BP and 2DG for a better therapeutic outcome. It is expected that such a combined strategy by concurrently blocking exogenous and endogenous pathways of nutrition supplys may be highly informative to the future design of tumor‐specific nanotherapies.

## Conflict of Interest

The authors declare no conflict of interest.

## Supporting information

Supporting Information
