# Supplementary material for: Augmenting Tumor‐Starvation Therapy by Cancer Cell Autophagy Inhibition
Source: Adv Sci (Weinh). 2020 Jan 27;7(6):1902847. doi: 10.1002/advs.201902847 (PMC7080508; doi:10.1002/advs.201902847)
Supplement: Supplementary file 1 — Supporting Information [file ADVS-7-1902847-s001.pdf]

Copyright WILEY-VCH Verlag GmbH & Co. KGaA, 69469 Weinheim, Germany, 2020.

ADVANCED SCIENCE
Open Access

Supporting Information

for *Adv. Sci.,* DOI: 10.1002/advs.201902847

Augmenting Tumor-Starvation Therapy by Cancer Cell Autophagy Inhibition

Bowen Yang, Li Ding, Yu Chen, and Jianlin Shi**

Copyright WILEY-VCH Verlag GmbH & Co. KGaA, 69469 Weinheim, Germany, 2020.

Supporting Information

Augmenting Tumor-Starvation Therapy by Cancer Cell Autophagy Inhibition

Bowen Yang[†]*, Li Ding*[†]*, Yu Chen* and Jianlin Shi**

Dr. Bowen Yang, Dr. Li Ding, Prof. Yu Chen and Prof. Jianlin Shi
State Key Laboratory of High Performance Ceramics and Superfine Microstructure, Shanghai Institute of Ceramics, Chinese Academy of Sciences, Shanghai, 200050, P. R. China.
E-mail: chenyu@mail.sic.ac.cn; jlshi@mail.sic.ac.cn

Dr. Bowen Yang, Prof. Yu Chen and Prof. Jianlin Shi
Center of Materials Science and Optoelectronics Engineering, University of Chinese Academy of Sciences, Beijing, 100049, P.R. China.

Dr. Bowen Yang and Dr. Li Ding contributed equally to this work.

A: Experimental Section

Preparation of BP nanosheets. 40 mg of BP powder (Jiangsu XFNANO Materials Tech Co., Ltd.) was added into 80 mL of *N*-methyl-2-pyrrolidone (NMP, Shanghai Macklin Biochemical Co., Ltd.), followed by an ice-bath probe sonication for 3 h at 1800 W. Then, the resultant brown suspension was centrifuged at 7000 rpm for 20 min, and the supernatant containing BP nanosheets was decanted gently.

Surface modification of BP nanosheets. The obtained BP nanosheets suspension was centrifuged at 20000 rpm for 20 min to remove NMP, and the precipitate was washed with deionized water for several times. Subsequently, 1 mg of BP nanosheets and 5 mg of PEG-NH_2 (MW 1000, Shanghai Yare Biotech, Co., Ltd.) were dispersed in 5 ml of deionized water, followed by a bath sonication for 30 min and a magnetic stirring for 8 h. Then, the mixture was centrifuged at 13,000 rpm for 20 min to remove excessive PEG-NH_2, and the precipitate was washed with deionized water for two times. The resultant pure PEGylated BP nanosheets were resuspended in deionized water or PBS for further use. In the following section, the word BP nanosheets is used to refer to PEGylated BP nanosheets for a simplified elucidation, unless otherwise specified.

Characterization. TEM image, FFT image and inverse fast FFT images were acquired on JEM-2100F electron microscope (operated at 200 kV). SEM image was obtained on field-emission Magellan 400 microscope (FEI Company). Size and Zeta potential measurements were conducted on Zetasizer Nanoseries (Malvern, UK). AFM image and Raman spectra were obtained on in Via (Renisha, UK). XPS was performed on ESCAlab250 (Thermal Scientific). FTIR spectra were recorded by Nicolet iS 10 spectrometers (Thermo Scientific). The concentration of BP nanosheets was measured by inductively coupled plasma optical emission spectrometer (ICP-OES, Agilent Technologies).

Cell culture. Human malignant melanoma cell line A375, human cervical cancer cell line HeLa and human umbilical vein endothelial cells (HUVECs) were purchased from the cell bank of the Chinese Academy of Sciences. The two cell lines were cultured in DMEM (high glucose, Gibco, USA) and supplemented with 10 % fetal bovine serum (FBS, Gibco, USA), and 1% penicillin/streptomycin (Gibco, USA) in a humidified incubator (5% CO_2 at 37 °C).

Glycolysis analysis. *Extracellular lactate content analysis.* A375 or Hela cells were seeded in 96-well plates (Corning, USA) at an initial density of 1 × 10^4 cells/well for 24 h, then treated with different concentrations of 2DG (Shanghai Bidde Pharmaceutical Technology Co., Ltd., from 0, 2, 4, 8, 16, 32 to 64 mM), BP nanosheets (0, 2, 4, 8, 16, 32 and 64 ppm) or their mixture dispersed in culture medium for 24 h and 48 h, respectively. The supernatants were obtained and the Lactate Assay Kit-WST® (Dojindo, L256) was used to quantify the extracellular lactate content. The linear addition of the effects of single 2DG and BP on lactate content was calculated following the equation (1):

$$[Lactate]_{Linear\ addition,x,x} = 1 - \left[\left(1 - \overline{[Lactate]_{2DG,x}}\right) + \left(1 - \overline{[Lactate]_{BP,x}}\right)\right] \quad (1)$$

Where $[Lactate]_{Linear\ addition,x,x}$ represents the linear addition of the effects of x mM of 2DG and x ppm of BP nanosheets on extracellular lactate content, $\overline{[Lactate]_{2DG,x}}$ is the average value of lactate content after the treatment of x mM of 2DG, $\overline{[Lactate]_{BP,x}}$ is the average value of lactate content after the treatment of x ppm of BP.

Intracellular ATP analysis. A375 or Hela cells were seeded in 6-well plates (Corning, USA) at an initial density of 2 × 10^5 cells/well for 24 h, then treated with 2DG (64 mM), BP nanosheets (64 ppm) or their mixture (64 mM of 2DG and 64 ppm of BP) dispersed in culture medium for 24 h and 48 h, respectively. Cells treated with pure culture medium was set as control. Then, the culture mediums were discarded and the ATP Assay Kit (Beyotime,

Shanghai, China) was used to quantify intracellular ATP level. The linear addition of the effects of single 2DG and BP on ATP level was also calculated following the equation (2):

$$[ATP]_{Linear\ addition,x,x} = 1 - \left[\left(1 - \overline{[ATP]_{2DG,x}}\right) + \left(1 - \overline{[ATP]_{BP,x}}\right)\right] \quad (2)$$

Where $[ATP]_{Linear\ addition,x,x}$ represents the linear addition of the effects of x mM of 2DG and x ppm of BP nanosheets on intracellular ATP content, $\overline{[ATP]_{2DG,x}}$ is the average value of ATP content after the treatment of x mM of 2DG, $\overline{[ATP]_{BP,x}}$ is the average value of ATP content after the treatment of x ppm of BP. In this experiment, x is 64.

Immunoblotting. A375 or HeLa cells were incubated in 6-well plates. After the indicated treatments, cells were washed with PBS for 3 times, followed by the addition of 100 ml of lysis buffer (Sigma, X100) and a proteinase inhibitor cocktail (Roche, 11873580001). Protein was collected at 4 °C, and the concentration was determined using a BCA Protein Assay Kit (Beyotime, P0009). Each sample was mixed with an equal amount of proteins, which was transferred to polyvinylidene difluoride B (PVDF) membranes after being separated by SDS-PAGE (Beyotime, P0012AC). The PVDF membranes were blocked with 5% nonfat dry milk at room temperature for 1 h, and then incubated with antibodies against GLUT1 (Cell Signaling Technology, 12939S), HK2 (Cell Signaling Technology, 2106), MYC (Cell Signaling Technology, 9402), ULK1 (Cell Signaling Technology, 6439), LDHA (Cell Signaling Technology, 3582) and GAPDH (Cell Signaling Technology, 5174) overnight. Then the films were washed for 3 times. PVDF membranes were incubated with horseradish peroxidase (HRP)-labeled secondary antibodies for 45 min, and then ECL reagent (Pierce, 32132) was added and allowed to react for 5 min. The films were exposed in a darkroom, and an imaging system (New Life Science Products, Boston, MA, USA) was used for visualization. The experiment was repeated 3 times. The protein expressions were quantified by software Image J.

Autophagy analysis. *Confocal laser scanning microscopy.* A375 or HeLa cells were seeded in 15-mm glass bottom cell culture dishes (NEST, 801002) with a density of 2 × 10^5 cells/dish and allowed to adhere overnight. Then Lipofectamine^{TM} 2000 transfection reagent (Invitrogen, 11668019) was applied to transfect pEGFP-LC3 (National Institute for Basic Biology, Okazaki, Japan) into the two cell lines (6 h of incubation). Subsequently, the medium was discarded, and fresh culture mediums containing different processing factors were added into corresponding culture dishes. After 24 h of incubation, the medium was also discarded and Lyso-Tracker Red (Beyotime, C1046) was used to stain the lysosomes. Finally, cells were

rinsed with PBS for 3 times and observed using FV1000 confocal fluorescence microscope (Olympus Company, Japan).

Immunoblotting. A375 or HeLa cells were incubated in 6-well plates. After the indicated treatments, cells were washed and lysed, and the protein was collected. The PVDF membranes were blocked with 5% nonfat dry milk at room temperature for 1 h, then incubated with antibodies against beclin 1 (Cell Signaling Technology, 3738), LC3B (Cell Signaling Technology, 3868S), Atg5 (Cell Signaling Technology, 2630), Atg12 (Cell Signaling Technology, 2010), p62 (Cell Signaling Technology, 16177) and GAPDH (Cell Signaling Technology, 5174) overnight. Then the films were washed for 3 times. PVDF membranes were incubated with HRP-labeled secondary antibodies for 45 min, and ECL reagent was added and allowed to react for 5 min. The films were exposed in a darkroom and observed. The experiment was repeated 3 times. The protein expressions were quantified by software Image J.

Flow cytometry. A375 or HeLa cells were seeded in 6-well plates. 0.05 mM of DAPGreen (Dojindo, D676) was added to the culture medium, followed by 30 min of incubation. Then, cells were rinsed with PBS for 3 times, and fresh culture mediums containing different processing factors were added into corresponding plates subsequently. After 24 h of incubation, cells were rinsed with PBS for 3 times and detected using a flow cytometer (BD FACSCalibur, Franklin Lakes, New Jersey, USA).

Bio-TEM. A375 or HeLa cells were seeded in 10-cm plates (Corning, USA) at an initial density of 1×10^6 cells/plate. After 24 h of incubation, cells were treated with 2DG (64 mM), BP nanosheets (64 ppm) or their mixture (64 mM of 2DG and 64 ppm of BP) for another 24 h. Cells treated with pure culture medium was set as control. Subsequently, cells were trypsinized, washed and resuspended in 2% glutaraldehyde fixative (Sigma, G5882), then embedded in 2% agarose, post-fixed in osmium tetroxide, and dehydrated with an acetone series. The obtained samples were further infiltrated and embedded in resin and polymerized at 60 °C for 24 h. The semithin sections were made into ultrathin sections, counterstained with 3% uranyl acetate and 0.3% lead citrate, and then observed on JEM-2100F electron microscope.

Intracellular pH analysis. A375 or Hela cells were seeded in 96-well plates at an initial density of 1×10^4 cells/well. After 24 h of incubation, cells were treated with different concentrations of BP nanosheets (0, 8, 16, 32 and 64 ppm) dispersed in culture medium. After another 24 h of incubation, the culture mediums were discarded and the pH probe 2',7'-bis-

(2-carboxyethyl)-5-(and-6)-carboxyfluorescein, acetoxymethyl ester (BCECF AM, Beyotime, S1006) was used to evaluate the intracellular pH. A panel of HUVECs was also treated with different concentrations of BP nanosheets for comparison.

Apoptosis assay. *Cell viability evaluation.* A375 or HeLa cells were seeded in 96-well plates at an initial density of 1×10^4 cells/well for 24 h, then treated with different concentrations of 2DG (0, 2, 4, 8, 16, 32 and 64 mM), BP nanosheets (0, 2, 4, 8, 16, 32 and 64 ppm) or their mixture dispersed in culture medium. After 12 h or 24 h of incubation, the culture mediums were discarded and Cell Counting Kit-8 assay (CCK-8, Dojindo, CK04) was used to evaluate the cell viabilities.

Flow cytometry. The death pathways of A375 or Hela cells was analyzed by the fluorescein isothiocyanate (FITC)-labeled Annexin V and propidium iodide (PI) double-staining method. In brief, A375 or Hela cells were seeded in 6-well plates for 24 h, then treated with 2DG (64 mM), BP nanosheets (64 ppm) or their mixture (64 mM of 2DG and 64 ppm of BP) for another 24 h. Cells treated with pure culture medium was set as control. Subsequently, cells were washed with PBS for 2 times, then an appropriate amount of 0.25 % trypsin solution (Gibco, USA) was added to each well for cell harvesting. The collected medium was diluted with DMEM containing 10 % FBS, followed by centrifugation for cell collection. According to the provided instructions, 2×10^5 cells were suspended in 0.5 mL of binding solution, and the Annexin V-FITC/PI Apoptosis Detection Kit (Dojindo, AD10) was added to each tube. The cells were allowed to stain in the dark at room temperature for 15 min. Then, the cells were immediately analyzed by flow cytometer.

Immunoblotting. A375 or HeLa cells were incubated in 6-well plates. After the indicated treatments, cells were washed and lysed, and the protein was collected. The PVDF membranes were blocked with 5% nonfat dry milk at room temperature for 1 h, then incubated with antibodies against cleaved caspase 3 (Cell Signaling Technology, 9661) and GAPDH (Cell Signaling Technology, 5174) overnight. Then the films were washed for 3 times. PVDF membranes were incubated with HRP-labeled secondary antibodies for 45 min, and ECL reagent was added and allowed to react for 5 min. The films were exposed in a darkroom and observed. The experiment was repeated 3 times. The protein expressions were quantified by software Image J.

Xenograft tumor model. 4-week old Female Balb/c nude mice (~15 g) were obtained from Charles River Laboratories (Beijing). Animal experiment procedures were confirmed to the guidelines for the Animal Care Ethics Commission of Shanghai Tenth People's Hospital,

Tongji University School of Medicine. Xenografted tumors were established by sub-cutaneous injection of 2 × 10^6 A375 or HeLa cells resuspended in 100 μL of DMEM into the mice and allowed to establish. The tumor size was measured by a digital caliper every two days and tumor volume (V) was calculated according to the following formula:

$$V_x = \frac{L_x \times W_x^2}{2} \tag{3}$$

Where L,W represent the length and width of tumor after x-day treatment.

The relative tumor volume (R) was calculated following the equation (4):

$$R_x = \frac{V_x}{V_0} \times 100\% \tag{4}$$

Antineoplastic effects. When the tumor size reached about 100 mm^3, A375 or HeLa tumor-bearing nude mice were randomly divided into 4 groups. 50 μL of PBS or the same dose of PBS containing 0.15 μg 2DG, 25 μg BP nanosheets or their mixture were intratumorally injected. Tumor volume and body weight were measured every two days, and the survival time of rats after various treatments was recorded. The linear addition of the effects of monotherapies on relative tumor volume was also calculated following the equation (5):

$$R_{Linear\ addition,x} = \overline{R_{Control,x}} - \left[\left(\overline{R_{Control,x}} - \overline{R_{2DG,x}}\right) + \left(\overline{R_{Control,x}} - \overline{R_{BP,x}}\right)\right] \tag{5}$$

Where $R_{Linear\ addition,x}$ represents the linear addition of the effects of 2DG and BP nanosheets on relative tumor volume after x days of treatment, $\overline{R_{Control,x}}$ is the average value of relative tumor volumes in control groups after x days of treatment, $\overline{R_{2DG,x}}$ is the average value of relative tumor volumes in 2DG groups after x days of treatment, $\overline{R_{BP,x}}$ is the average value of relative tumor volumes in BP groups after x days of treatment.

The antineoplastic effect of different experimental groups was also calculated following the equation (6):

$$A_G = \frac{\left(\overline{R_{Control,14}} - 100\%\right) - \left(\overline{R_{G,14}} - 100\%\right)}{\left(\overline{R_{Control,14}} - 100\%\right)} \tag{6}$$

Where G indicates 2DG, BP or synergistic therapy groups, A_G represents the antineoplastic effect of 2DG, BP or synergistic therapy groups, $\overline{R_{Control,14}}$ is the average value of relative tumor volume in control groups at day 14, $\overline{R_{G,14}}$ is the average value of relative tumor volume in 2DG, BP or synergistic therapy groups at day 14.

Histology. *Efficacy evaluation.* After 14 days of treatments, the tumor-bearing mice were euthanized and xenograft tumor tissues were harvested. Tissues were washed with PBS and immersed in 4% paraformaldehyde (PFA) for histological analysis. For immunohistochemical examination, the tumor sections were incubated with antibodies against Ki67 (Cell Signaling Technology, 9027), LDHA (Cell Signaling Technology, 3582), LC3B (Cell Signaling Technology, 3868S) and p62 (Cell Signaling Technology, 16177) at 4 °C overnight, followed by treatment with biotinylated secondary antibody. These antibody complexes were detected using an ABC Kit (Vector Laboratories, CA, USA). For immunofluorescent evaluation, the tumor sections were incubated with antibodies against LC3B (Cell Signaling Technology, 3868S) and GLUT1 (Cell Signaling Technology, 12939S), followed by treatment with biotinylated secondary antibody. DNA was stained with TUNEL and 4',6-diamidino-2-phenylindole (DAPI). These sections were observed using FV1000 confocal fluorescence microscope.

Biosafety evaluation. The heart, liver, spleen, lungs and kidneys were collected from the euthanized tumor-bearing mice at day 14 and fixed in 4% PFA. Then tissue sections were stained with H&E. The stained sections were observed using light microscopy (Olympus IX51, Tokyo, Japan).

Statistical analysis. Data was analyzed by SPSS 20 statistical software. Two-sided *t*-testing was used in this work for comparison. The data was indicated with n.s. for not significant, * for probability less than 0.05 ($P < 0.05$, significant), ** for $P < 0.01$ (moderately significant), and *** for $P < 0.001$ (highly significant), respectively.

B: Supplementary Figures

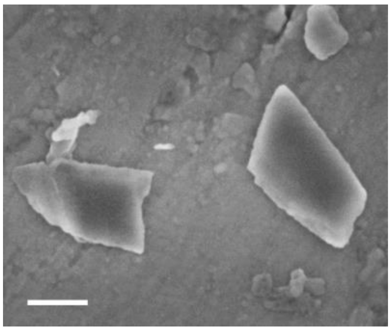

Figure S1. SEM image of prepared BP nanosheets. Scale bar, 50 nm.

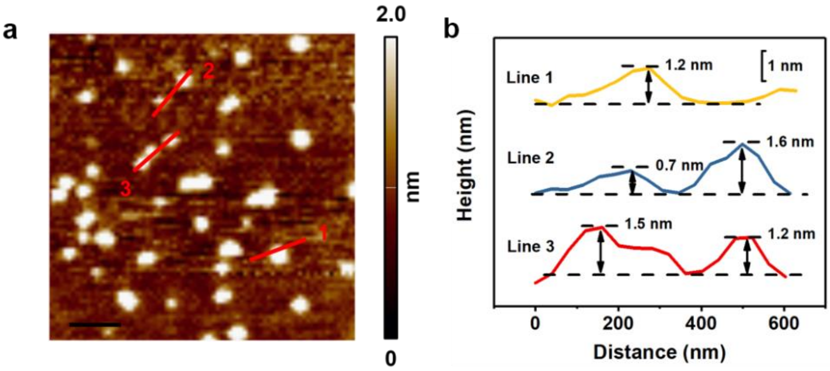

Figure S2. AFM analysis. **(a)** AFM image of highly-dispersed BP nanosheets. Scale bar, 400 nm. **(b)** Thickness measurement of BP nanosheets based on the three red lines in (a).

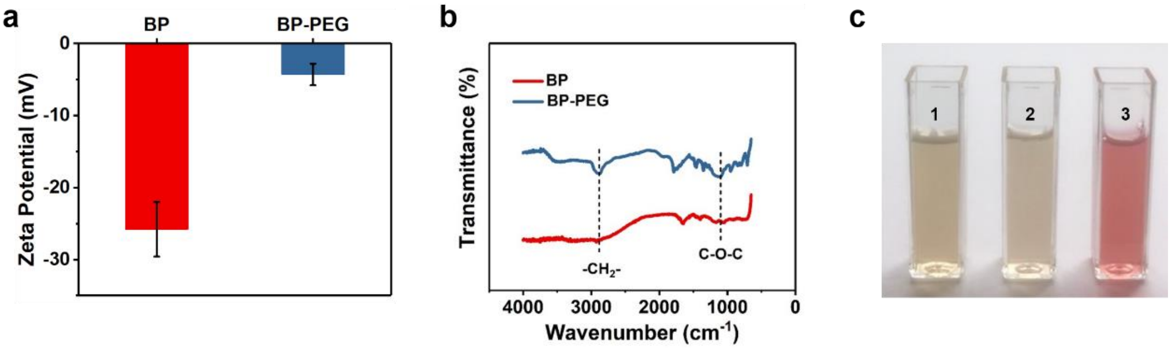

Figure S3. Surface modification of BP nanosheets. **(a)** Zeta potentials of BP nanosheets before and after PEGylation. Error bars are based on standard deviation (SD, N = 3). **(b)** FTIR spectra of BP nanosheets before and after PEGylation. **(c)** Digital photographs of PEGylated BP nanosheets dispersed in H_2O (1), PBS (2) and DMEM (3) for 2 h.

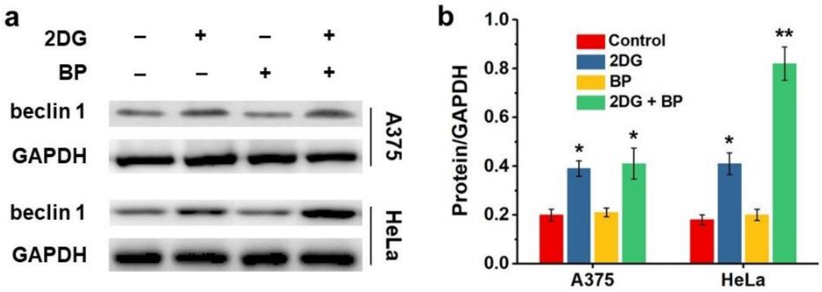

Figure S4. Immunoblot analyses of beclin 1 expression levels in A375 and HeLa cells after different treatments for 24 h. GAPDH expression levels serve as the loading controls. Experiments were repeated three times with similar results. Statistical significances were calculated via Student's *t* test. *$P < 0.05$ and **$P < 0.01$.

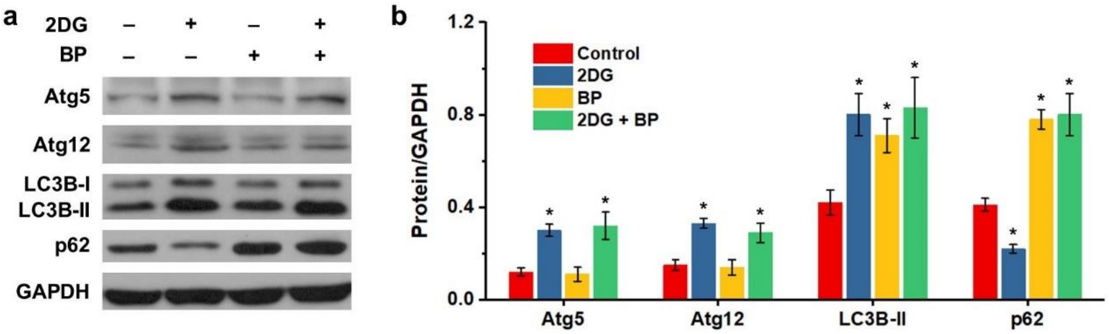

Figure S5. Immunoblot analyses of Atg5, Atg12, LC3B and p62 expression levels in HeLa cells after different treatments for 24 h. GAPDH expression levels serve as the loading controls. Experiments were repeated three times with similar results. Statistical significances were calculated via Student's *t* test. *$P < 0.05$.

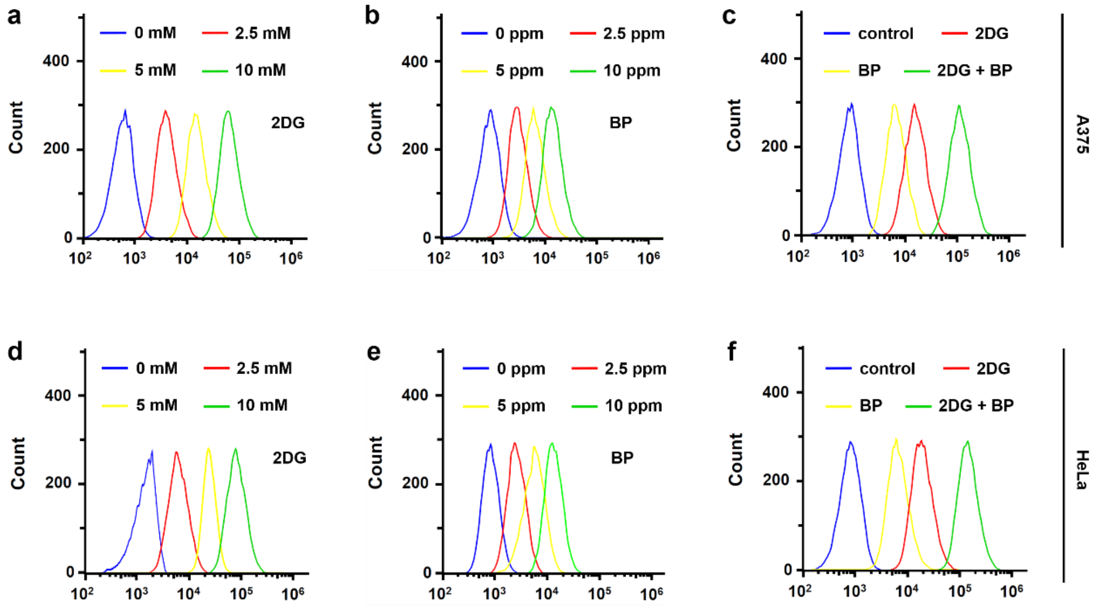

Figure S6. Flow cytometric autophagic analysis of DAPGreen-stained cancer cells. A375 (a-c) and HeLa (d-f) cells were incubated with different concentrations of 2DG (a, d) or different concentrations of BP nanosheets (b, e) or different types of processing factors (c, f).

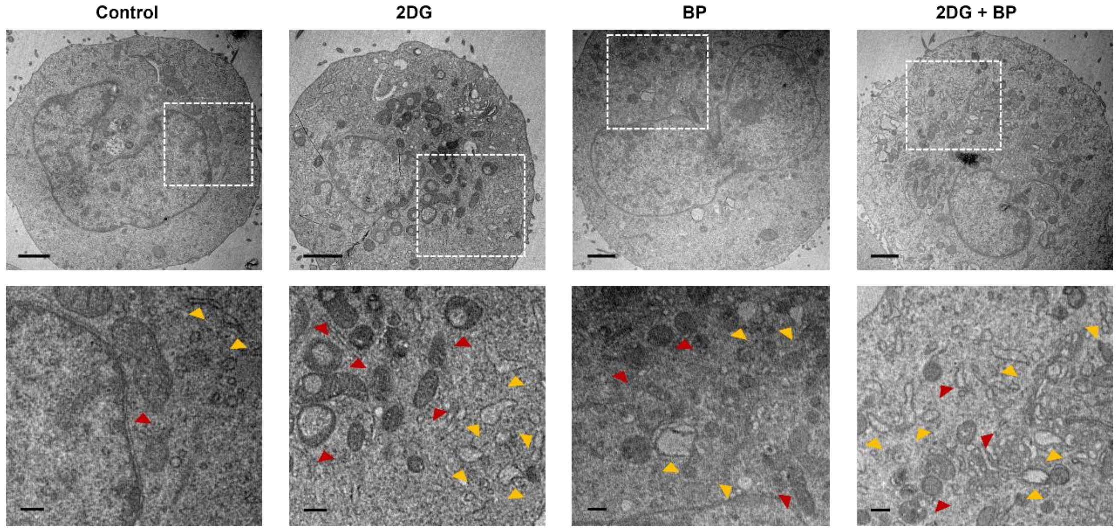

Figure S7. Bio-TEM images of HeLa cells after various treatments. Yellow and red triangle marks represent initial and degradative autophagic vacuoles, respectively. Scale bars, up row, 2 μm; down row, 0.5 μm.

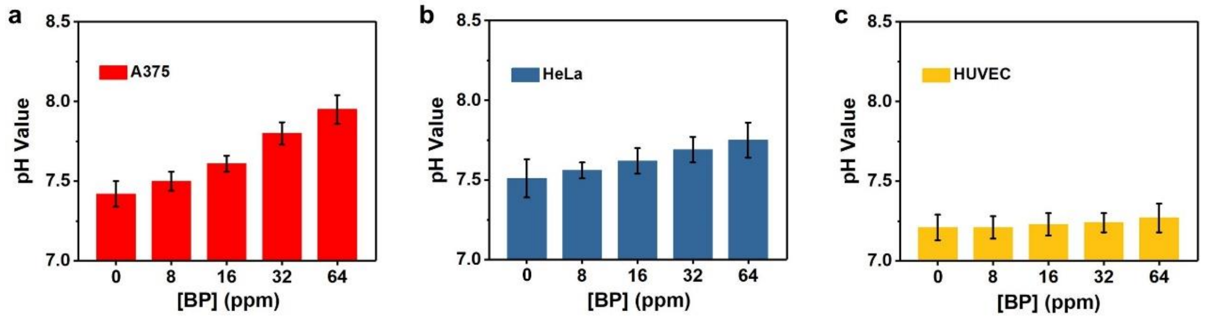

Figure S8. Changes of intracellular pH of A375 cells **(a)**, HeLa cells **(b)** and normal cell line HUVECs **(c)** after treatments with increasing concentrations of BP nanosheets for 24 h. Error bars are based on SD (N = 6). Although it is acknowledged that the tumor microenvironment is characterized with mild acidity (extracellular pH < 7.1),[1,2] the intracellular pH of cancer cells is around 7.4.[2] Comparatively, the intracellular pH of normal cells is around 7.2.[2] The addition of BP nanosheets promotes the generation of alkaline PO_4^{3-} in cancer cells after reacting with intracelluar ROS, which depletes H^+ from H_2O and finally elevates the intracellular pH.

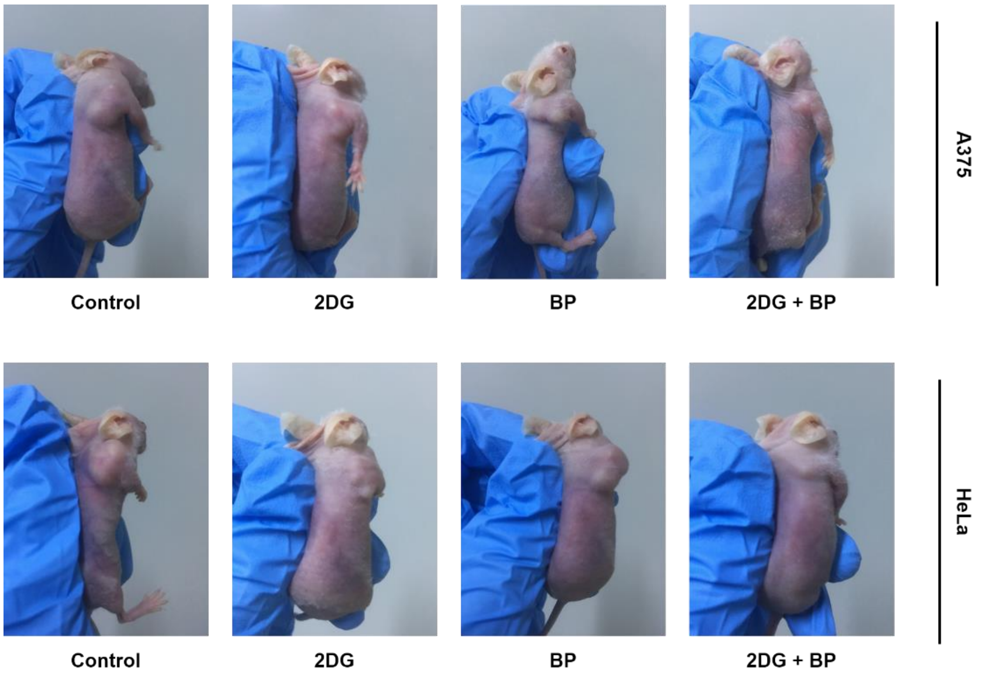

Figure S9. Photographs of A375 and HeLa tumor-bearing mice after different treatments at day 14. The growth of tumors in 2DG and BP groups was slightly restrained, while efficient inhibition on tumor growth could be observed in synergistic therapy groups.

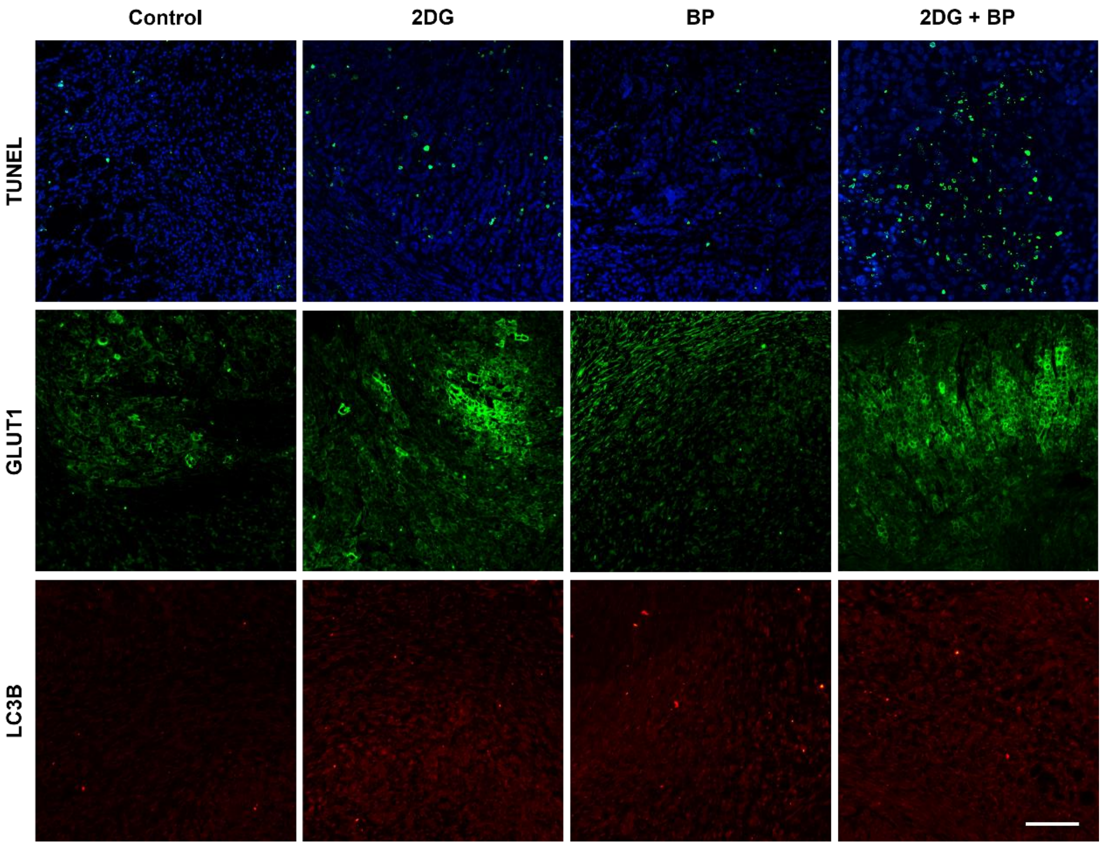

Figure S10. Immunofluorescence analyses of TUNEL, GLUT1 and LC3B of xenografted A375 tumor sections after different treatments at day 14. Blue fluorescence represents cell nuclear stained with DAPI. Scale bars, 100 μm.

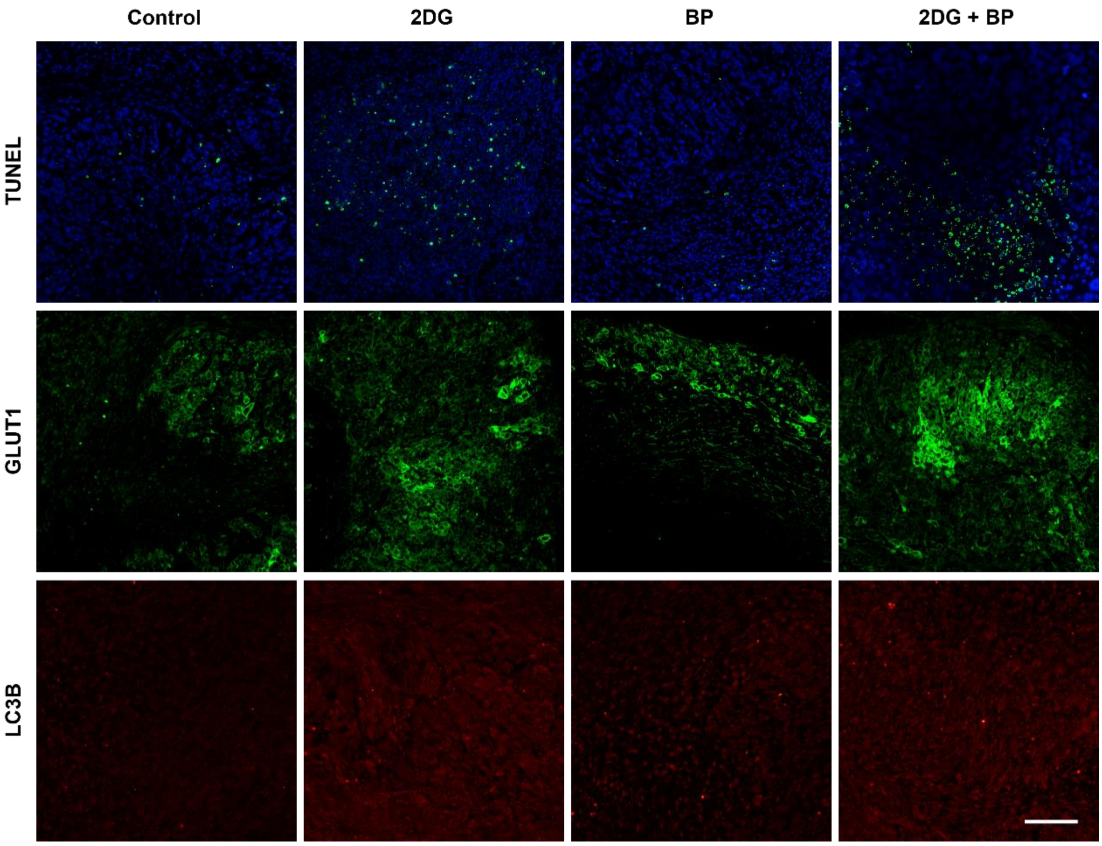

Figure S11. Immunofluorescence analyses of TUNEL, GLUT1 and LC3B of xenografted HeLa tumor sections after different treatments at day 14. Blue fluorescence represents cell nuclear stained with DAPI. Scale bars, 100 μm.

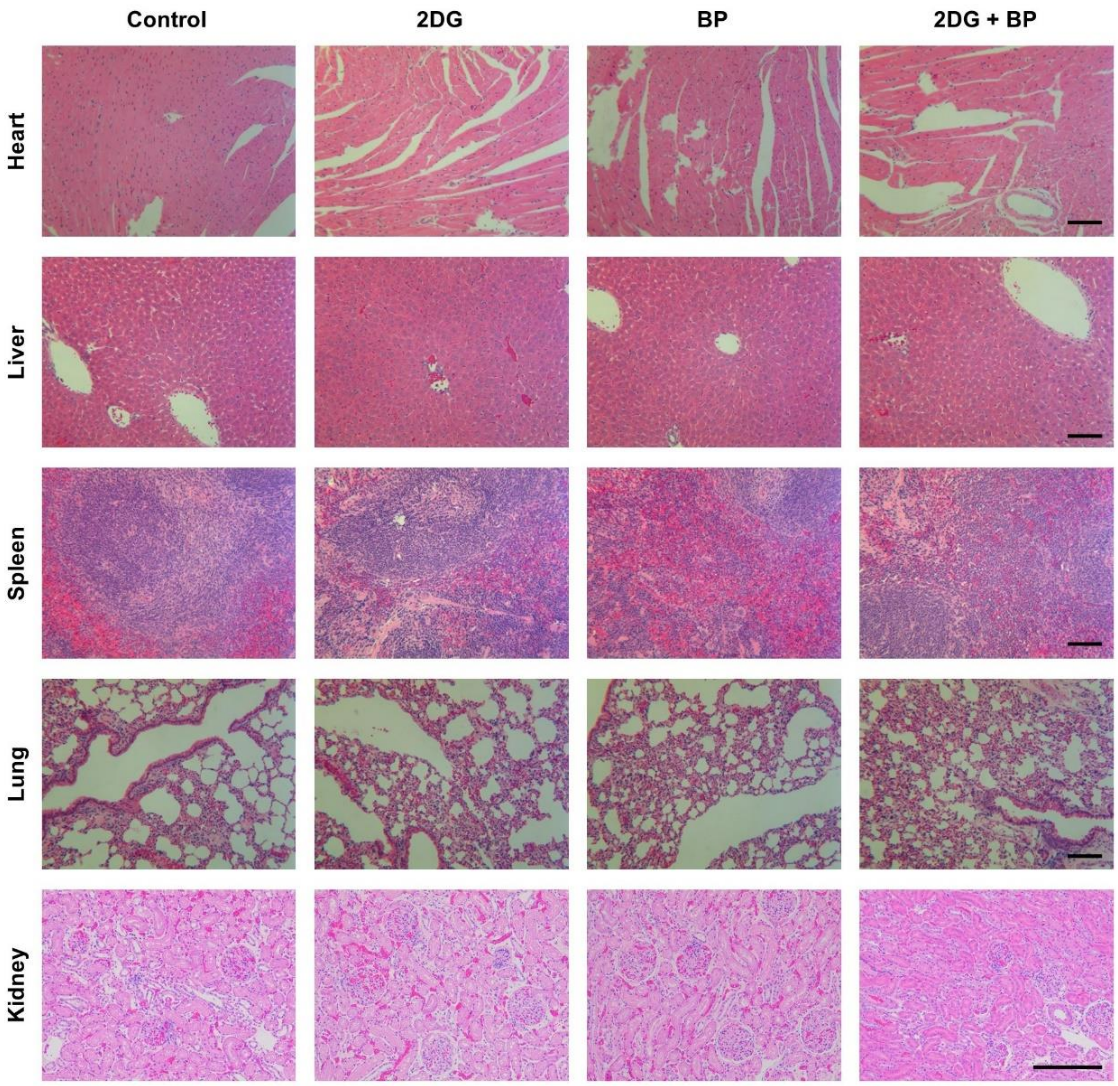

Figure S12. H&E staining of major organs (heart, liver, spleen, lung, and kidney) harvested from A375 tumor-bearing mice after different treatments at day 14. Scale bars, 100 μm.

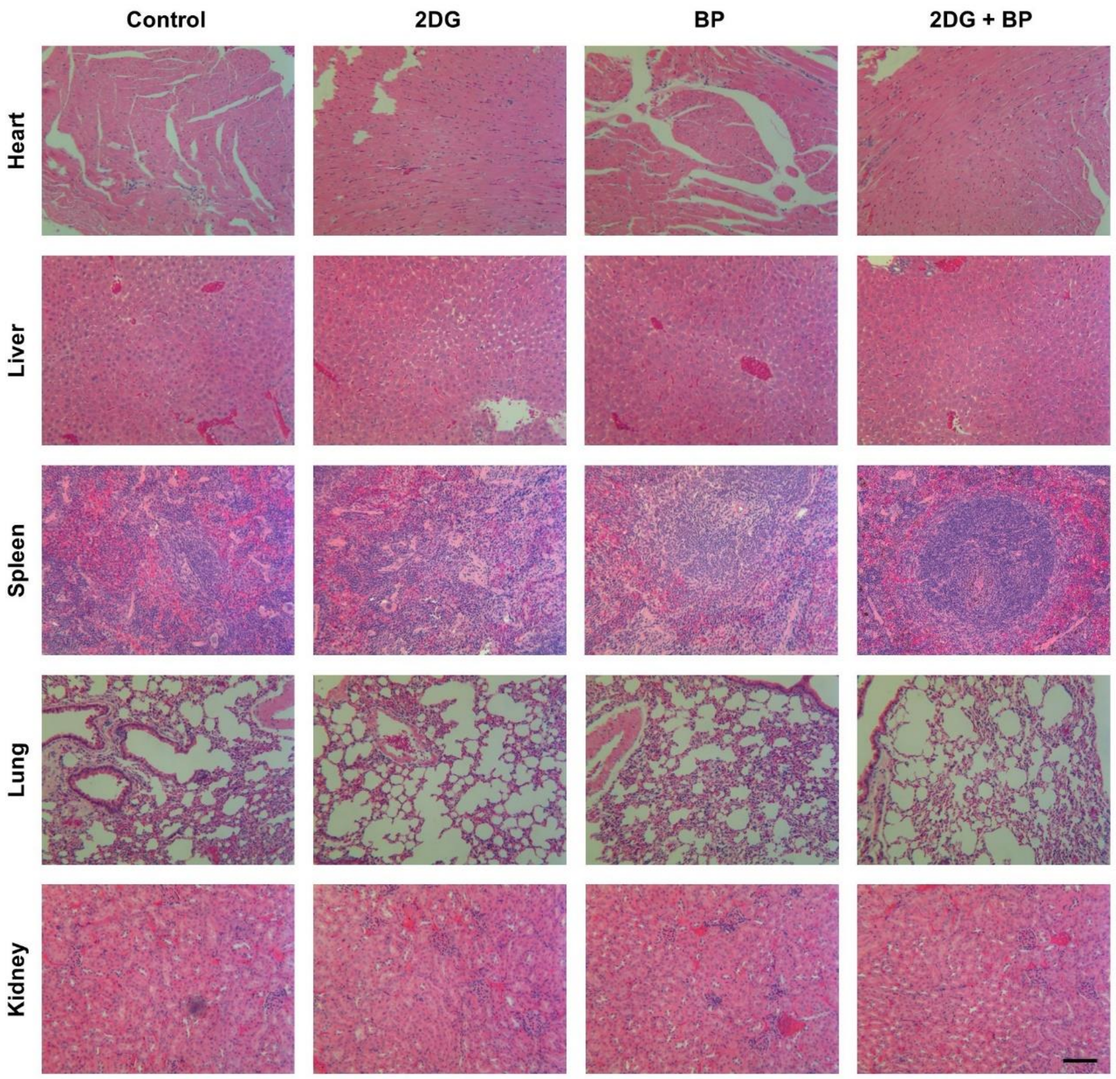

Figure S13. H&E staining of major organs (heart, liver, spleen, lung, and kidney) harvested from HeLa tumor-bearing mice after different treatments at day 14. Scale bars, 100 μm.

C: Supplementary References

[1] V. Estrella, T. Chen, M. Lloyd, J. Wojtkowiak, H. H. Cornnell, A. Ibrahim-Hashim, K. Bailey, Y. Balagurunathan, J. M. Rothberg, B. F. Sloane, J. Johnson, R. A. Gatenby, R. J. Gillies, *Cancer Res.* **2013**, *73*, 1524.

[2] B. A. Webb, M. Chimenti, M. P. Jacobson, D. L. Barber, *Nat. Rev. Cancer* **2011**, *11*, 671.
